# Stem Cell-Derived Corneal Epithelium: Engineering Barrier Function for Ocular Surface Repair

**DOI:** 10.3390/ijms26157501

**Published:** 2025-08-03

**Authors:** Emily Elizabeth Fresenko, Jian-Xing Ma, Matthew Giegengack, Atalie Carina Thompson, Anthony Atala, Andrew J. W. Huang, Yuanyuan Zhang

**Affiliations:** 1 Wake Forest Institute for Regenerative Medicine, School of Medicine, Wake Forest University, Winston-Salem, NC 27101, USA; emily.fresenko@wfusm.edu (E.E.F.);; 2 School of Medicine, Wake Forest University, Winston-Salem, NC 27101, USA; 3 Department of Biochemistry, School of Medicine, Wake Forest University, Winston-Salem, NC 27101, USA; 4 Department of Ophthalmology, School of Medicine, Wake Forest University, Winston-Salem, NC 27101, USA; 5 Department of Ophthalmology and Visual Sciences, Washington University School of Medicine, St. Louis, MO 63110, USA; huangandrew@wustl.edu

**Keywords:** corneal reconstruction, stem cells, epithelium, barrier function, regenerative medicine

## Abstract

The cornea, the transparent anterior window of the eye, critically refracts light and protects intraocular structures. Corneal pathologies, including trauma, infection, chemical injury, metabolic diseases, genetic conditions, and age-related degeneration, can lead to significant visual impairment. While penetrating keratoplasty or full-thickness corneal transplantation remains a standard and effective intervention for severe corneal dysfunction, limitations in donor tissue availability and the risk of immunogenic graft rejection necessitate alternative therapeutic strategies. Furthermore, for cases of isolated epithelial disfunction, a full-thickness cornea graft may not be required or effective. This review examines the potential of corneal epithelial constructs derived from autologous stem cells with functional barrier properties for corneal reconstruction and in vitro pharmacotoxicity testing. In this review, we delineate the current limitations of corneal transplantation, the advantages of stem cell-based approaches, and recent advances in generating engineered corneal epithelium. Finally, we address remaining technical challenges and propose future research directions aimed at clinical translation.

## 1. Introduction

The cornea, the transparent anterior portion of the eye, plays a dual role in ensuring optical clarity and providing a protective barrier to environmental threats [1]. Its curvature and avascular nature contribute to approximately 70% of the eye’s total refractive power, enabling sharp image focus on the retina [2]. This optical performance is largely attributed to the cornea’s organized stromal structure and dehydrated state, maintained by endothelial ion transport [2].

Beyond optics, the cornea functions as an immunological and physical shield, actively protecting the intraocular structures from a myriad of environmental insults. These include harmful ultraviolet (UV) radiation, a wide spectrum of pathogenic microorganisms (bacteria, viruses, fungi), airborne particulate matter, and chemical irritants [3]. The cornea’s multilayered structure is exquisitely adapted for these functions, comprising five distinct layers—the superficial epithelium, Bowman’s layer, the thick central stroma, Descemet’s membrane, and the innermost endothelium—each contributing to clarity and protection [1,2]. Among these layers, the corneal epithelium, as the outermost layer, is the first line of defense and holds a remarkable capacity for regeneration in response to injury [4].

However, despite its inherent regenerative capabilities, the corneal epithelium remains acutely vulnerable to a range of insults. These include acute trauma, chemical and thermal burns, pervasive infections (bacterial keratitis, herpetic keratitis), autoimmune disorders (e.g., ocular cicatricial pemphigoid, Stevens–Johnson syndrome), and various genetic conditions (e.g., aniridia-associated keratopathy) [5]. When an injury involves the limbal region—where the corneal stem cells reside—the native regenerative capacity of the epithelium may be severely compromised or even destroyed. This leads to a debilitating condition known as limbal stem cell deficiency (LSCD) [4,6]. LSCD includes conjunctivalization (the aberrant invasion of conjunctival goblet cells and vascular tissue onto the typically avascular cornea), neovascularization (ingrowth of blood vessels), and chronic, non-healing epithelial defects. Clinically, LSCD manifests as profound pain, severe photophobia, recurrent infections, and ultimately significant and often irreversible vision loss [6].

While corneal transplantation (penetrating keratoplasty) remains the current gold-standard treatment for severe corneal injury and opacification, its broader implementation and long-term success are significantly hampered by two limitations: (1) donor tissue shortage and (2) allograft rejection [7,8]. LSCD is untreatable with penetrating keratoplasty, as the epithelial layer post-penetrating keratoplasty comes from the recipients’ own limbal stem cells. While limbal stem cell grafts can be performed, they are much more likely to suffer allograft rejection, as the limbus is vascularized while the rest of the cornea is not. These challenges underscore the urgent and unmet clinical need for novel regenerative strategies that are capable of effectively restoring corneal epithelial barrier function, achieving durable transparency, and ideally obviating the reliance on limited donor tissue and chronic systemic immunosuppression [4].

Stem cell-derived corneal epithelium has emerged as a highly promising and transformative alternative [9,10,11]. A diverse array of stem cell types, including resident limbal stem cells (LSCs), multipotent mesenchymal stem cells (MSCs), ethically sensitive but highly pluripotent embryonic stem cells (ESCs), and patient-specific induced pluripotent stem cells (iPSCs) [12,13,14,15], possess the remarkable capacity to generate stratified epithelial sheets in vitro [4,9,16]. When appropriately differentiated and engineered, these constructs can robustly recapitulate the native barrier function of the limbus region of the cornea and actively promote healing of the ocular surface [17,18,19]. Beyond their direct therapeutic applications in tissue repair, these precisely engineered epithelial constructs also offer invaluable in vitro platforms for pharmacotoxicity screening of novel ophthalmic drugs and for developing sophisticated disease models that enable a deeper understanding of corneal pathologies at the cellular and molecular levels.

This review aims to provide a comprehensive overview of the current state of the art in stem cell-derived corneal epithelia. We will specifically focus on the cutting-edge engineering strategies employed for their generation, the rigorous methods used for functional evaluation of their barrier properties, and the crucial translational progress that is bringing these therapies closer to the clinic. Furthermore, we will summarize recent advances in stem cell sourcing, optimized differentiation protocols, and advanced tissue engineering approaches while concurrently identifying key challenges that still need to be overcome and outlining future opportunities for successful clinical application.

## 2. Limbal Stem Cell (LSC) Implantation for Corneal Repair in Clinical Practice

Limbal stem cell (LSC) transplantation is a cornerstone in the surgical management of limbal stem cell deficiency (LSCD), a debilitating ocular surface disorder characterized by chronic pain, inflammation, and vision loss. The primary objective is to restore a stable, transparent corneal epithelium by replenishing functional LSCs. The choice of transplantation technique is guided by the laterality (unilateral vs. bilateral) and severity (partial vs. total) of LSCD (Table 1).

### 2.1. Autologous Limbal Stem Cell Transplantation (for Unilateral LSCD)

In unilateral LSCD, the contralateral healthy eye can serve as an autologous donor, mitigating risks of immune rejection and obviating the need for systemic immunosuppression [20,21]. Conjunctival limbal autograft (CLAU) involves harvesting 2–4 clock hours of limbal tissue with adjacent conjunctiva from the healthy eye, followed by direct transplantation to the affected eye post-pannus removal [20,21]. CLAU is a single-stage procedure with long-term safety and efficacy, although there is a risk of iatrogenic LSCD in the donor eye due to substantial tissue harvest. Anatomical success rates up to 83.2% have been reported [21]. To address the concerns regarding donor morbidity and limited tissue quantity, cultivated autologous limbal epithelial transplantation (CLET) uses a much smaller limbal biopsy (1–2 mm^2^) from the healthy eye, which is expanded ex vivo on a biological substrate such as denuded human amniotic membrane under good manufacturing practice (GMP) conditions [16,20]. Once a confluent epithelial sheet has formed, it is transplanted onto the recipient cornea. CLET minimizes donor tissue harvesting, allows for quality control of proliferative cells, and has reported success rates ranging from 60% to 80% for restoring a stable, avascular corneal surface [16,20]. However, CLET is a two-stage procedure requiring specialized cell culture facilities, increasing complexity and cost compared to direct grafting [20,23]. A newer single-stage alternative, simple limbal epithelial transplantation (SLET), combines the minimal donor tissue requirement of CLET with the simplicity of CLAU. SLET involves harvesting a small limbal biopsy (2–3 mm^2^) from the healthy eye, mincing it into tiny explants, and placing them directly onto the periphery of the de-epithelialized recipient cornea, often secured with fibrin glue and covered with an amniotic membrane. SLET has gained popularity due to its cost-effectiveness, reduced donor morbidity, and reported success rates of 75.2% to 83.8% at one year [20,23,24] (Table 1).

### 2.2. Allogeneic Limbal Stem Cell Transplantation (for Bilateral LSCD)

In bilateral LSCD, autologous sources are unavailable, necessitating the use of allogeneic (donor-derived) LSCs, which introduces challenges of allograft rejection and the need for systemic immunosuppression [7,15,23]. Keratolimbal allograft (KLAL) and living-related conjunctival limbal allograft (lr-CLAL) involve transplantation of limbal tissue from a cadaveric donor or living relative [20,21]. While these procedures provide LSCs in the absence of autologous options, they require lifelong systemic immunosuppression, leading to significant systemic side effects and high risk of immune rejection [7,23,25]. KLAL graft survival rates have been reported at 67% at 12 months and 53% at 18 months [16]. Allogeneic cultivated limbal epithelial transplantation is similar to autologous CLET, but uses donor-derived LSCs cultured ex vivo [21,23]. This cultured donor epithelium is potentially less immunogenic than full tissue grafts, but still requires immunosuppression and may suffer from limited long-term survival of transplanted cells, with some studies reporting anatomical success rates of 61.4% and functional success rates of 53% [23,24] (Table 1).

### 2.3. Key Clinical Considerations and Challenges for All LSC Transplantation

Several clinical considerations are critical for successful LSC transplantation. Meticulous preoperative optimization of the ocular surface is essential and includes control of inflammation, management of dry eye, correction of eyelid abnormalities, and resolution of cicatricial complications [15,20,23]. Confirmation of LSCD through impression cytology to detect goblet cells on the cornea is crucial for accurate diagnosis and appropriate treatment [10,15]. Persistent inflammation poses a significant threat to LSC survival and function; thus, effective anti-inflammatory strategies are vital before, during, and after surgery. Long-term follow-up is necessary to monitor for graft stability, recurrence of LSCD, and complications such as glaucoma (particularly with corticosteroid use), infection, or progressive corneal or conjunctival scarring [7,20,26]. The absence of unequivocal markers for LSCs complicates diagnosis and assessment of transplanted cell “stemness,” highlighting the need for ongoing research into reliable LSC biomarkers. For ex vivo expanded cell therapies such as CLET, strict good manufacturing practice (GMP)-compliant manufacturing and quality control protocols are essential to ensure cell viability, purity, and safety [16,22].

In summary, LSC implantation has transformed the treatment landscape for LSCD. Autologous approaches, particularly CLAU, CLET, and SLET, offer the most promising and durable outcomes for unilateral LSCD. In bilateral cases, allogeneic transplantation remains the mainstay, despite the burden of lifelong immunosuppression and higher failure rates. These challenges underscore the need for continued research into novel regenerative solutions and immune-evasive therapies for LSCD [9,20,23,25].

## 3. Limitations of Corneal Transplantation

Despite its widespread use as a sight-restoring procedure, conventional corneal transplantation (penetrating keratoplasty) faces significant limitations, particularly in cases involving corneal scarring, primary epithelial dysfunction, or severe limbal stem cell deficiency (LSCD). These challenges underscore the pressing need for innovative regenerative strategies [7,20,23].

### 3.1. Donor Tissue Shortage

Relatively few limbal stem cell grafts are being performed in the United States and worldwide. This is not because there is not a need, but rather because of the greater difficulty of LSC graft versus full thickness and endothelial keratoplasty. As methods of limbal stem cell grafting become more available, these procedures will be potentially subject to the same tissue availability issues as other corneal transplants. A major and persistent hindrance to widespread corneal transplantation is the global shortage of suitable donor corneas [27,28]. This tissue scarcity means that many patients in need of transplantation face long waiting lists, potentially leading to irreversible tissue damage or vision loss. Exacerbating this chronic issue, events such as the COVID-19 pandemic have profoundly impacted tissue procurement and distribution, leading to drastic declines in available donor corneas—by as much as 68% in some regions during peak pandemic periods [20,29]. This critical scarcity has intensely catalyzed research into alternative solutions aimed at circumventing the reliance on limited donor tissue. Such innovative approaches include the robotic fabrication of epithelial sheets, which offers potential for automated, high-throughput production, and the development of low-density cell cultures derived from minimal donor tissue, maximizing the yield from each available donor [14,16]. Notably, groundbreaking research has demonstrated the ability to engineer stratified epithelial sheets from even a single adult murine limbal progenitor cell [16], presenting a high-yield strategy that could revolutionize tissue engineering and dramatically increase the availability of transplantable tissue.

### 3.2. Immune Rejection

Corneal graft rejection remains a critical and frequently encountered challenge following transplantation, representing a leading cause of graft failure. This risk is particularly pronounced in high-risk patients (e.g., those with prior graft failures, extensive corneal vascularization, or severe ocular surface inflammation), where the failure rates due to rejection can tragically reach as high as 35–70% [7,20,30]. Rejection is particularly an issue for limbal stem cell grafts, as the limbus is vascularized and lacks the immune privilege that the rest of the cornea is equipped with. Rejections can manifest from distinct immunological outcomes affecting different layers of the cornea, including epithelial rejection lines, characterized by a line of lymphocytes migrating across the epithelium; endothelial rejection lines, often visible as a “Khodadoust line” of inflammatory cells on the posterior corneal surface; and a more diffuse stromal rejection band, indicative of deeper inflammation and commonly seen in penetrating keratoplasties [7,31,32]. Accompanying anterior chamber reactions, marked by intraocular inflammation, and severe corneal edema resulting from endothelial dysfunction can ultimately lead to opacification of the previously clear graft and significant vision loss.

To mitigate rejection, corticosteroids are commonly prescribed as systemic or topical immunosuppressants. However, long-term use of corticosteroids carries significant risks [7,26], including elevated intraocular pressure (leading to glaucoma), cataract formation, and increased susceptibility to infections, thereby introducing secondary complications that can further jeopardize visual outcomes. To address these profound risks, alternative strategies are continuously emerging. These include the development of biomaterial-free cell sheets, which minimize foreign material exposure, and the exploration of anti-inflammatory agents such as IL-1 receptor antagonists, which can locally suppress immune activation and promote endogenous stem cell expansion, potentially leading to a more tolerant ocular surface [33,34,35]. Importantly, successful ocular surface reconstruction depends not only on the regeneration of the epithelial layer but also on the functional integrity of the underlying corneal stroma and endothelium. The epithelium cannot sustain itself in isolation: it relies on biochemical and structural support from the stromal matrix and the hydration-regulating function of the endothelium to maintain transparency and stability. Therefore, effective biointegration of the epithelial graft or carrier with the host stroma is critical to ensure epithelial cell survival, differentiation, and long-term tissue homeostasis [36,37].

Together, these inherent challenges of donor tissue scarcity and the persistent threat of immune rejection underscore the urgent and compelling need for regenerative strategies capable of durably restoring corneal integrity while simultaneously reducing the reliance on limited donor tissue and eliminating the necessity for lifelong systemic immunosuppression.

## 4. Advantages of Autologous Stem Cell-Derived Epithelium

Autologous stem cell-derived corneal epithelium offers a compelling array of advantages over conventional transplantation approaches, which often rely on donor cadaveric tissue [13,14,29]. These significant benefits largely stem from the inherent regenerative potential of stem cells, their reduced immunogenicity in an autologous setting, and the unprecedented capacity for precise customization through advanced tissue engineering techniques [16,29,33].

### 4.1. Unlimited Renewal Capacity

A fundamental advantage of stem cells is their inherent ability for self-renewal, allowing for the theoretical generation of a virtually unlimited supply of patient-specific corneal epithelial cells [16,38]. This addresses the critical issue of donor tissue scarcity, which is a major bottleneck in conventional corneal transplantation [23]. Limbal stem cells (LSCs), physiologically residing at the limbus (the crucial junction between the cornea and conjunctiva), are the primary source responsible for maintaining corneal epithelial homeostasis and repair throughout life [16,39]. In cases of limbal stem cell deficiency (LSCD), where this resident population is compromised, restoring epithelial integrity becomes challenging [7]. However, when LSCs are expanded ex vivo from a small biopsy or when pluripotent stem cells, such as induced pluripotent stem cells (iPSCs) or embryonic stem cells (ESCs), are guided to differentiate into corneal epithelial cells, an abundant and renewable source becomes available [13,14,29]. This unlimited supply ensures that multiple applications or follow-up procedures are feasible without relying on external donors, revolutionizing the scalability of treatment [16]. Nevertheless, the process of inducing LSCs from various autologous sources can be arduous and time-consuming [40].

### 4.2. Directed Epithelial Differentiation

Stem cells possess the remarkable plasticity to be guided to differentiate into stratified, cornea-specific epithelial cells through tightly regulated and optimized in vitro protocols. This controlled differentiation process leverages our understanding of corneal development [13,16]. Key transcription factors, such as p63, Pax6, and KLF4, play orchestrating roles in managing lineage commitment and the establishment of the corneal epithelial phenotype [16,41]. As cells mature, specific cytokeratins (e.g., cytokeratin 3 (K3) and cytokeratin 12 (K12)) serve as definitive markers of mature, differentiated corneal epithelium, confirming successful lineage specification [17,42]. By meticulously fine-tuning culture conditions, including the precise composition of growth factors, cytokines, and small molecules in the media, as well as the mechanical and biochemical properties of the substrate (e.g., stiffness, surface coatings), these stem cells can be engineered to accurately replicate the native tissue’s complex cellular architecture, vital optical transparency, and crucial barrier functions [13,43,44]. This directed differentiation ensures the generation of functionally competent tissue rather than just a simple sheet of cells.

### 4.3. Reduced Immunogenicity and Rejection Risk

Perhaps one of the most compelling advantages of using autologous stem cell-derived epithelium is the near elimination of immunogenicity and the associated risk of immune rejection. Unlike allogeneic (donor-derived) grafts, which inherently express foreign major histocompatibility complex (MHC) antigens and other surface molecules that trigger a robust host immune response, autologous cells are recognized as “self.” This bypasses the need for chronic systemic immunosuppression, a critical benefit as long-term immunosuppression carries significant risks of opportunistic infections, nephrotoxicity, and malignancy [7,9,18,33,34]. While some stem cell types, like mesenchymal stem cells (MSCs), possess intrinsic immunomodulatory properties that can dampen immune responses, the differentiated cells derived from allogeneic stem cells will inevitably express MHC antigens and other surface molecules that can elicit an immune response [33,34]. Therefore, autologous applications fundamentally provide the most reliable and safest strategy for achieving long-term engraftment and avoiding immune-mediated graft failure [13,14,35]. This dramatically improves patient safety and quality of life post-transplantation.

### 4.4. Customization Through Tissue Engineering

The integration of stem cell biology with advanced tissue engineering principles allows for unprecedented customization of corneal substitutes [16,43]. Biomaterial-based scaffolds, which serve as the structural framework for the engineered tissue, can be precisely designed and seeded with stem cell-derived epithelium [19,44]. These scaffolds can be tailored for specific attributes, including their mechanical strength (to withstand eye movements and surgical handling), optical transparency (crucial for vision), and their ability to integrate seamlessly with the host corneal tissue [43,45]. The profound ability to control the cellular microenvironment (e.g., oxygen tension, nutrient delivery) and meticulously tailor biomaterial properties (e.g., porosity, degradation rate, biochemical cues) offers a significant advantage over the fixed properties of donor corneas [13,14]. Moreover, differentiation strategies used to prepare the epithelial layer can significantly influence functional outcomes, including tight junction formation and barrier integrity. Studies have shown that improved differentiation protocols lead to higher TEER values, as demonstrated in a microfluidic co-culture cornea-on-a-chip model where TEER exceeded 650 Ω·cm^2^, and increased expression of tight junction proteins, suggesting closer mimicry of native corneal epithelium [46]. Similarly, scaffold material properties such as pore size, stiffness, and surface chemistry have been shown to directly impact epithelial cell adhesion, proliferation, and differentiation—factors critical for robust barrier function. These outcomes are often quantified through TEER and permeability assays, and several scaffold-based studies have reported such metrics to validate physiological relevance [47,48,49]. By directing stem cell differentiation, proliferation, and their organized spatial arrangement on these scaffolds, corneal substitutes can potentially be engineered with superior optical clarity, enhanced biomechanical integrity, and optimized functional integration with host tissues, leading to potentially better visual outcomes than traditional grafts [29,50].

### 4.5. Trophic Support

**Figure 1:**
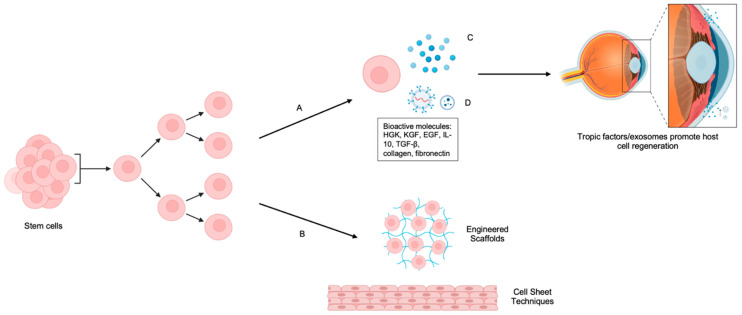
Strategies for stem cell therapy for corneal injury. Stem cells can be induced to differentiate into corneal lineages through exposure to specific differentiation cues. Two major therapeutic strategies are depicted: (Arrow A) secretion-based therapy, in which stem cells release trophic factors (C) and extracellular vesicles (EVs) (D)—including exosomes and microvesicles—rich in bioactive molecules (e.g., HGF, KGF, EGF, IL-10, TGF-β, collagen, and fibronectin). EVs facilitate tissue repair by delivering regulatory cargo such as miRNAs, mRNAs, proteins, and lipids to host corneal cells, promoting epithelial proliferation, anti-inflammatory responses, and extracellular matrix remodeling. (Arrow B) Cell-based replacement therapy, where engineered scaffolds or cell sheet techniques are used to transplant stratified, stem cell-derived epithelial structures onto damaged corneal surfaces. Created with BioRender.com accessed on 16 June 2025.

Beyond their ability to differentiate and form a structural barrier, stem cells exert significant trophic effects on the surrounding tissue [34,35]. This involves the secretion of a rich array of bioactive molecules, including various growth factors (e.g., HGF, KGF, EGF), cytokines (e.g., IL-10, TGF-β), chemokines, and extracellular matrix components (e.g., collagen, fibronectin) [18,51]. These secreted factors collectively modulate inflammation by suppressing pro-inflammatory mediators (e.g., IL-1β, TNF-α), thereby creating a more favorable microenvironment for healing [33,52]. They also actively promote epithelial repair by stimulating endogenous cell proliferation and migration, and notably support neural regeneration [34,53] (Figure 1). This neurotrophic support is vital for restoring corneal sensation, which is critical for reflex tearing and protecting the ocular surface [28,50]. Furthermore, these secreted factors contribute to overall corneal surface stability and the maintenance of a healthy tear film, both essential for long-term graft success and patient comfort. This paracrine signaling capability of stem cells adds an invaluable dimension to their therapeutic potential, going beyond simple tissue replacement [18,52].

## 5. Progress in the Field

Significant advancements have been made in stem cell-based corneal reconstruction, with progress spanning multiple stem cell sources, differentiation techniques, and scaffold innovations [14,29,43].

### 5.1. Multiple Stem Cell Sources

**Figure 2:**
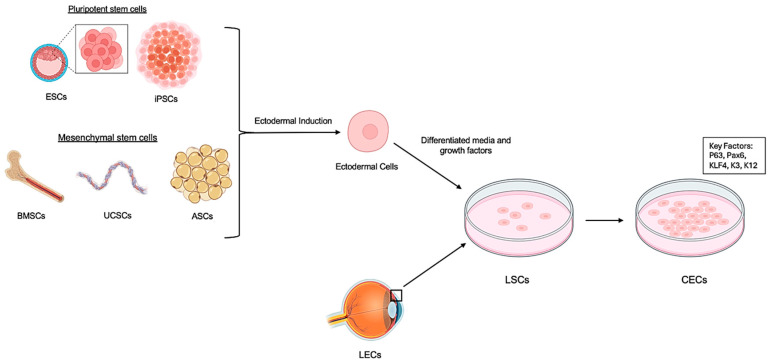
Stem cell isolation and culture. Both pluripotent stem cells—including embryonic stem cells (ESCs) and induced pluripotent stem cells (iPSCs)—and mesenchymal stem cells, such as bone marrow-derived stem cells (BMSCs), umbilical cord stem cells (UCSCs), and adipose-derived stem cells (ASCs), can undergo ectodermal induction to generate ectodermal cells. These ectodermal cells, when exposed to specific differentiation media and growth factors, can be directed to form limbal stem cells, which further differentiate into corneal epithelial cells. Alternatively, native limbal epithelial cells (LECs) isolated from the ocular surface can serve as a direct source for corneal epithelial cell production. Created with BioRender.com accessed on 16 June 2025.

Various stem cell types have been explored for their regenerative potential in corneal repair (Table 2). These include embryonic stem cells (ESCs), induced pluripotent stem cells (iPSCs), mesenchymal stem cells (MSCs), limbal stem cells (LSCs), and adipose-derived stem cells (ASCs) [13,14,40,53]. ESCs have demonstrated robust differentiation into corneal epithelial phenotypes, especially when cultured on acellular porcine corneal matrices [42,54]. iPSCs offer personalized, expandable sources, and have been used to create 3D corneal organoids [55]. MSCs, derived from umbilical cord blood and peripheral blood mononuclear cells, show homing ability and therapeutic efficacy in injured corneal tissues [18,56]. Other sources, such as umbilical cord lining stem cells and oral mucosal epithelial cells, have shown promise for long-term integration and epithelial repair [51,56] (Figure 2, Table 2).

### 5.2. Optimized Differentiation Protocols

Protocols have been standardized for differentiating stem cells into corneal epithelial cells (CECs) and limbal stem cells (LSCs) with proper morphology and functional characteristics. These protocols leverage key growth factors, extracellular matrices, and feeder cell systems [16,42,43]. For instance, Springer Protocols provides a standardized method for generating corneal epithelium from cultures of keratinocytes, fibroblasts, and corneal epithelial cells derived from both human and rabbit sources [32]. Human basal limbal epithelial cells have been isolated using BCAM-positive sorting techniques, enabling reproducible epithelial sheet fabrication [40]. Advances include feeder-free, oxygen-controlled culture methods and the use of limbal stem cell-conditioned media to induce limbal-like phenotypes from hESCs on porcine scaffolds [24,41,48,54] (Table 3).

### 5.3. Stratified Epithelium Derived from Stem Cells for Corneal Regeneration

Generating a functional, stratified corneal epithelium is crucial for restoring visual clarity [14]. LSCs, CESCs, iPSCs, MSCs, and ESCs have all been investigated for this potential [40,51,53,55]. While ex vivo LSC expansion shows clinical promise, issues such as phenotype maintenance and donor availability remain. iPSCs and ESCs provide scalable alternatives, but pose risks of teratoma formation and immunogenicity [58,63]. Successful stratification also depends on a scaffold choice (e.g., amniotic membranes, collagen gels, and decellularized stroma) and the presence of key growth factors to drive differentiation [21,43,44,64].

Together, these innovations in stem cell sourcing, differentiation, and epithelium engineering have advanced the field toward viable clinical applications in corneal repair and regeneration/replacement [18,52].

## 6. Key Functional Parameters of Barrier Function

**Figure 3:**
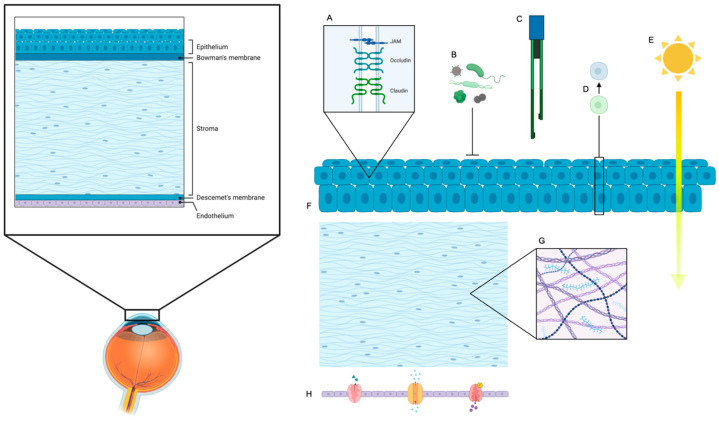
Corneal epithelial, stromal, and endothelial cell functional aspects. A schematic representation of the human cornea highlighting its five main layers: epithelium, Bowman’s membrane, stroma, Descemet’s membrane, and endothelium. The epithelium forms the outermost barrier via tight junctions (**A**), defending against pathogens (**B**), maintaining transepithelial electrical resistance (**C**), enabling limbal stem cell (LSC) differentiation into corneal epithelial cells (**D**), optimizing light transmission (**E**), and organizing its surface from the limbus (**F**). Stromal keratocytes (**G**) preserve corneal structure through extracellular matrix (ECM) turnover and support epithelial homeostasis. Corneal endothelial cells (**H**) control stromal hydration via ion transport, ensuring corneal transparency. Created with BioRender.com accessed on 16 June 2025.

A critical and paramount goal in engineering stem cell-derived corneal epithelium is to ensure its functional integrity precisely mimics the native corneal barrier. This barrier is indispensable for maintaining corneal transparency, hydration, and protecting the inner ocular structures from external insults. To rigorously assess the physiological competence and barrier performance of regenerated tissue, several well-defined parameters are employed, each contributing a distinct and crucial insight (Figure 3). Importantly, these functional metrics are directly influenced by the scaffold material properties and stem cell differentiation protocols discussed earlier, emphasizing the interconnectedness of design choices and biological outcomes.

### 6.1. Transepithelial Electrical Resistance (TEER)

Transepithelial electrical resistance (TEER) is a widely recognized and quantitative electrophysiological measurement that assesses the integrity of tight junctions within an epithelial cell monolayer [47,65]. It directly quantifies the ionic resistance across the epithelial layer, making it a primary and highly sensitive indicator of barrier tightness [66]. Higher TEER values are indicative of stronger tight junction integrity, meaning a more robust seal between adjacent cells and consequently significantly reduced paracellular permeability [31,67]. This tight sealing is essential for maintaining the precise hydration balance of the cornea, preventing excessive fluid influx or efflux, and, critically forming a physical barrier against the infiltration of pathogens, such as bacteria and viruses, from the external environment. TEER measurements are typically performed using chopstick electrodes or Endohm chambers, providing real-time, non-destructive monitoring of barrier development and maturation in cultured epithelial sheets [7]. This functional outcome reflects the impact of scaffold stiffness and differentiation methods on tight junction formation and barrier integrity.

### 6.2. Molecular Permeability

Molecular permeability testing evaluates the selective permeability of the epithelial barrier by quantifying the passage of various tracer molecules across the engineered tissue [68,69]. This is typically achieved using fluorescently labeled dextrans of different molecular weights (e.g., 4 kDa, 10 kDa, 40 kDa) or small-molecule drugs [10,70]. By measuring the flux of these tracers from one side of the epithelial monolayer to the other, researchers can determine the degree of restriction imposed by the barrier. Functional epithelium should exhibit a highly restricted permeability, particularly to larger molecules, while allowing for the passage of smaller, necessary ions and nutrients [71]. This selectivity is vital for nutrient exchange and maintaining the appropriate osmotic environment. A compromised barrier, conversely, would show increased permeability to larger molecules, indicating leaky tight junctions or cellular damage. This assay complements TEER by providing insight into the size-exclusion properties of the barrier [19]. These permeability characteristics have been linked to scaffold pore size and surface chemistry, underscoring the importance of scaffold design in achieving physiological barrier function.

### 6.3. Tight Junctions

Tight junctions (TJs) are multiprotein complexes located at the most apical aspect of the lateral cell membrane, forming a continuous seal around epithelial cells [72]. They are the primary structural components responsible for establishing and maintaining the epithelial barrier, regulating paracellular transport, and separating distinct apical and basolateral membrane domains. Key tight junction proteins include occludin, various claudins (e.g., claudin 1, claudin 3, claudin 7), and zonula occludens (ZO-1, ZO-2, ZO-3) proteins, which link the transmembrane proteins to the actin cytoskeleton [9,58]. Immunofluorescence microscopy is commonly used to visualize the continuous circumferential localization of these proteins at cell–cell borders, indicating proper tight junction formation [73]. Western blotting or qPCR can quantify the expression levels of these components. Proper localization and robust expression of these proteins are direct indicators of a functional tight junction network. Disrupted or mislocalized tight junctions are hallmarks of a compromised epithelial barrier [74]. The expression and organization of these proteins are enhanced by specific differentiation protocols and scaffold surface modifications, further demonstrating the interplay between biomaterial design and cellular function.

### 6.4. Fluid Regulation and Drug Penetration

The corneal epithelium plays a crucial physiological role in maintaining fluid homeostasis within the cornea, actively participating in water transport to regulate stromal hydration and transparency [75]. Furthermore, it acts as the primary barrier to the ocular penetration of topically applied drugs. Fluid transport studies, often involving the measurement of fluid movement across the epithelial sheet under defined osmotic or hydrostatic gradients, provide direct evidence of the tissue’s ability to regulate water balance [43]. Concurrently, in vitro permeability assays for ophthalmic drugs are conducted to assess how effectively the engineered epithelium allows or restricts the passage of therapeutic compounds [64,76]. This not only informs the regenerative success by demonstrating physiological functionality but also has significant implications for future therapeutic applications, guiding the formulation and delivery of ophthalmic medications [53,61]. A well-functioning epithelial barrier should allow for controlled drug penetration while maintaining its protective role. These functional capabilities are influenced by scaffold composition and structural properties, which affect epithelial differentiation and barrier tightness.

### 6.5. Crucial Role of Underlying Corneal Stroma and Endothelium in Ocular Surface Reconstruction

A successful and sustained ocular surface reconstruction hinges not only on the integrity of the epithelial layer but also, critically, on the health and functionality of the underlying corneal stroma and corneal endothelium. While the focus of this review is on the corneal epithelium, it is imperative to acknowledge that the epithelial layer cannot maintain its clarity or structural integrity in isolation.

The corneal stroma, comprising the bulk of the cornea, provides the essential structural support and contributes significantly to corneal transparency [9,54]. A healthy stroma is vital for proper epithelial adhesion and differentiation. Likewise, the corneal endothelium plays a pivotal role in maintaining corneal deturgescence, actively pumping fluid out of the stroma [75,77]. Dysfunction of either the stroma or the endothelium can lead to corneal edema, scarring, and ultimately vision loss [11,13], thereby compromising even a perfectly reconstructed epithelial surface.

Furthermore, the biointegration of the epithelial carrier with the residual corneal stroma is critical for epithelial survival and further differentiation. The close interplay between these layers ensures the necessary nutrient supply, waste removal, and biochemical signaling that are indispensable for the long-term viability and proper functioning of the newly reconstructed epithelial tissue [69]. Without robust biointegration, the epithelial graft may delaminate, leading to persistent defects and failure of the reconstructive effort. Therefore, strategies for ocular surface reconstruction must carefully consider and address the health and regenerative potential of the underlying corneal stroma and endothelium to achieve lasting success. This underscores the importance of scaffold design and biomaterial properties that promote not only epithelial function but also integration with stromal and endothelial layers.

### 6.6. The Emerging Role of Extracellular Vesicles in Corneal Reconstruction

The field of regenerative medicine has seen a surge of interest in extracellular vesicles (EVs), including exosomes and microvesicles, which are nanoscale lipid bilayer vesicles naturally released by cells [17,19,73,78]. These fascinating biological nanoparticles act as crucial mediators of intercellular communication by carrying a diverse cargo of proteins, lipids, messenger RNAs (mRNAs), and microRNAs (miRNAs) from their parent cells to recipient cells. Found abundantly in various biological fluids such as saliva, tears, urine, and blood, EVs possess remarkable potential for therapeutic applications due to their ability to transfer genetic information and bioactive molecules, influencing recipient cell behavior.

In the context of corneal reconstruction, EVs secreted by various stem cell populations—such as limbal stem cells, mesenchymal stem cells, and induced pluripotent stem cells—are gaining significant attention [10,79]. These stem cell-derived EVs can recapitulate many of the beneficial effects of their parent cells, including promoting cell proliferation, migration, and differentiation, while also exerting anti-inflammatory, anti-apoptotic, and pro-angiogenic effects [57,60]. For instance, studies have shown that EVs derived from mesenchymal stem cells can accelerate corneal epithelial wound healing, reduce corneal scarring, and promote nerve regeneration [11,14]. Their small size and natural membrane make them ideal carriers for targeted drug delivery to the ocular surface, potentially reducing systemic side effects [50,80].

The therapeutic utility of EVs in corneal reconstruction lies in their capacity to deliver specific biomolecules that can modulate the corneal microenvironment, suppress detrimental immune responses, and foster tissue repair [20,52]. This non-cellular approach offers several advantages over direct cell transplantation, including reduced immunogenicity, easier storage, and a lower risk of tumor formation. Further research into isolating, characterizing, and engineering these potent nanocarriers holds immense promise for developing novel, cell-free strategies to restore corneal clarity and function in patients suffering from severe corneal diseases. Integrating knowledge of EVs with scaffold design and stem cell differentiation approaches could further enhance functional outcomes and clinical efficacy.

These functional parameters—TEER, molecular permeability, tight junction analysis, and fluid/drug transport studies—serve as a rigorous and comprehensive framework for evaluating the overall barrier competence and physiological functionality of stem cell-derived corneal epithelium, stromal cells and endothelial cells, thereby guiding its continued development toward safe and effective clinical use. Finally, EVs play an important role in the development of optimal strategies of corneal repair.

## 7. Development of Biocompatible Scaffolds for Corneal Tissue Engineering

The strategic design of biocompatible scaffolds is paramount for supporting cell adhesion, proliferation, and differentiation in corneal tissue engineering [19,31]. These scaffolds serve not only as carriers for stem cell-derived epithelial sheets but also play a critical role in facilitating the seamless integration of engineered constructs with host corneal tissue [27,79]. An ideal scaffold should precisely mimic the native extracellular matrix, support stratified epithelial growth, maintain transparency, and allow for robust integration without provoking inflammation or fibrosis [33,53,60] (Figure 3). There are two types of biomaterials commonly used in corneal regeneration: natural and synthetic biomaterials. Each type has its own strengths and limitations (Table 3).

### 7.1. Natural Materials

Natural biomaterials are widely explored for their inherent biocompatibility and bioactivity, closely resembling the native corneal environment [43,62] (Table 3). Materials such as amniotic membrane, hyaluronic acid, collagen, chitosan, gelatin, and silk fibroin are commonly utilized [26,52,81]. Additionally, decellularized corneal tissue offers a truly biomimetic option, preserving the intricate architecture of the native cornea [82]. These materials effectively promote epithelial cell attachment, support cell proliferation, and help maintain native cellular morphology and function. However, their inherent properties often necessitate structural enhancement through cross-linking or combination with other materials to improve mechanical stability, control degradation rates, and ensure long-term integrity in vivo [25,70]. For instance, collagen and hyaluronic acid are crucial components of the corneal stroma and can be used to create hydrogels that support cellular growth and nutrient exchange [27,30].

### 7.2. Synthetic Polymers

Synthetic polymers offer tunable physical properties that are critical for creating scaffolds with precise characteristics [47,83] (Table 3). Materials like polyurethane, polycaprolactone (PCL), and polyethylene glycol (PEG)-based hydrogels allow for precise control over physical properties such as stiffness, elasticity, transparency, and degradation rate [77,78]. Their versatility enables customization to match the specific biomechanical and optical requirements of different layers of the cornea [17,61]. For example, porous PCL scaffolds can be engineered to match the curvature and thickness of the human cornea. While synthetic polymers offer excellent structural control, their bioactivity may need to be supplemented through surface functionalization with cell adhesion motifs (e.g., RGD peptides) or by serving as vehicles for the sustained delivery of growth factors and bioactive molecules [32,59].

### 7.3. Emerging Scaffold Technologies

Advanced scaffold designs increasingly combine the strengths of both natural and synthetic materials to leverage their synergistic properties [15,31]. This combinatorial approach allows for scaffolds that offer superior mechanical integrity, tunable degradation kinetics, and enhanced biological cues. Examples of such innovations include:

1. Electrospun polyurethane–silk nanofiber scaffolds: These composite scaffolds create a fibrous network that closely mimics the natural extracellular matrix, promoting robust epithelial regeneration and demonstrating improved mechanical strength [43,67].
2. Collagen membranes cross-linked with UV/riboflavin: This technique enhances the mechanical stability and enzymatic resistance of collagen membranes, providing improved support for epithelial growth and reducing premature degradation [9,84].
3. Porous hyaluronic acid hydrogels: The porous structure of these hydrogels significantly enhances nutrient diffusion throughout the scaffold, which is crucial for cell viability and metabolism, and improves compatibility with endothelial cells, which are highly sensitive to their microenvironment [67,68].
4. Cultured oral mucosal epithelial cell sheets, when applied in LSCD models, have demonstrated the remarkable ability to restore proteasome function, indicating significant potential for regenerative therapy by addressing cellular stress and protein degradation pathways [14,80].
5. 3D Bioprinting: This cutting-edge technology allows for the precise deposition of cells and biomaterials layer by layer, enabling the creation of highly complex and anatomically accurate corneal constructs with defined cellular arrangements and spatial control over growth factor delivery. This offers unprecedented control over scaffold architecture, moving towards truly personalized corneal grafts [11,73].
6. Smart and responsive scaffolds: Future developments include scaffolds that can respond to physiological cues, such as pH changes or enzymatic activity, to release therapeutic agents or degrade at a controlled rate, further optimizing the regenerative process [7,35].

As scaffold materials and fabrication technologies continue to evolve, the ability to construct fully functional, biointegrative corneal grafts becomes increasingly feasible [22,54]. These advancements are opening the door to the development of clinical-grade tissue-engineered alternatives, offering renewed hope for patients suffering from various forms of corneal disease.

## 8. Translational Research

Recent advances in stem cell-based therapies for corneal repair have successfully transitioned from preclinical studies to early-phase clinical trials, providing crucial proof-of-concept data for the safety and feasibility of engineered epithelial grafts [23,85]. A pioneering first-in-human trial at Osaka University Hospital evaluated the transplantation of allogeneic iPSC-derived corneal epithelial sheets in four patients with limbal stem cell deficiency (LSCD). Over a one-year follow-up, this study reported no graft-related adverse events and demonstrated improved corneal surface integrity in all patients [16,30].

Further clinical investigations include a trial of autologous mesenchymal stem cell transplantation (MSCT) in LSCD patients, which yielded outcomes comparable to cultivated limbal epithelial transplantation, without reported adverse events [41,42]. Additionally, cultivated autologous limbal epithelial cell (CALEC) transplantation was performed in 15 patients to assess a new manufacturing protocol and its efficacy. While one unrelated bacterial infection was observed, grafts successfully achieved partial or complete restoration of corneal surface integrity [9]. A comprehensive review of cultivated limbal epithelial transplantation (CLET) trials consistently confirms positive outcomes with long-term stability. This review underscores the advantages of ex vivo cell expansion under good manufacturing practice (GMP) conditions for minimizing immune rejection and pathogen transmission [23,40].

Moreover, mesenchymal stem cell-derived therapies continue to show significant promise. MSCs and their EVs exhibit the ability to modulate inflammation, promote epithelial healing, and reduce corneal neovascularization, positioning them as attractive candidates for cell-free or adjunctive approaches to traditional epithelial grafting [10,21,60]. These translational efforts collectively highlight the accelerating clinical momentum towards establishing safe, standardized, and scalable stem cell-based strategies for ocular surface reconstruction.

## 9. Challenges in Corneal Regeneration

Corneal regeneration offers significant promise for restoring vision lost to corneal damage. However, translating this potential into widespread clinical reality presents substantial challenges.

### 9.1. Overcoming Translational Hurdles

Standardized differentiation protocols are urgently needed to ensure the consistent generation of functional corneal epithelial tissue and accelerate clinical translation. These frameworks must comprehensively address safety concerns related to cell sourcing, manufacturing, quality control, and rigorous clinical trial design. Refining surgical techniques for delivering cells and tissues to the cornea is crucial to minimize trauma and maximize graft integration [31,85]. Complications such as infection, hemorrhage, and graft dislocation remain significant safety concerns. Therefore, standardization and comprehensive practitioner training are essential for procedural consistency and improved patient outcomes [7,20,23,63].

While allogeneic cell sources offer logistical advantages, they also carry immunogenic risks [86,87]. Immunological rejection remains a significant risk, even with autologous or HLA-matched cell sources [20,28]. The potential for teratoma formation, particularly with iPSC-derived cells, necessitates stringent quality control and differentiation protocols [69,70]. Long-term safety data for many emerging therapies are still lacking, requiring continued vigilance. The risk of pathogen transmission, especially with xenografts or certain donor-derived cells, demands rigorous safety screening [18,38]. Understanding and mitigating host immune responses will be critical, involving both immunosuppression protocols and tolerance-induction strategies. Xenotransplantation, despite offering an abundant cell source, introduces heightened immunogenicity and the potential for zoonotic disease transmission [41,80]. The choice of stem cell source—whether limbal stem cells, iPSCs, or mesenchymal stem cells—directly impacts therapeutic safety. Off-target differentiation, malignant transformation, and teratoma formation, particularly with pluripotent cells, must be rigorously assessed [53,55].

Scaffolds and carrier materials used in corneal regeneration must be biocompatible and effectively support tissue integration. Inflammatory reactions, degradation, or toxicity associated with biomaterials could jeopardize graft success, making rigorous preclinical evaluation of biomaterials critical [34,44]. Achieving consistent, functional, and refractive visual outcomes remains a major challenge. Simply replacing damaged corneal tissue is insufficient: regenerated tissue must integrate seamlessly with the host stroma and effectively restore optical clarity and refractive power [4,19]. Generating functional limbal stem cells (LSCs) with long-term viability is vital to maintaining corneal homeostasis [49,67]. Moreover, accurately recapitulating the cornea’s complex cellular architecture and extracellular matrix organization remains a substantial barrier. Seamless integration of the regenerated epithelium with host stroma is essential to avoid graft shrinkage, opacification, and disease recurrence.

Long-term studies are required to evaluate graft durability and monitor for delayed complications, including graft failure, cataracts, glaucoma, and recurrence of the original disease [42,88]. The influence of aging and systemic health on long-term graft function also warrants investigation. Finally, the economic burden of corneal regeneration therapies, stemming from the high costs of cell sourcing, culture, and transplantation, poses a significant barrier to accessibility [89]. Cost-reduction strategies, such as scalable manufacturing and the development of off-the-shelf therapies, are essential [60,90]. Comprehensive cost-effectiveness analyses will be crucial to validate these advanced therapies relative to conventional corneal transplantation [62].

### 9.2. Future Directions in Corneal Regeneration

Advancing the field of corneal regeneration hinges on several key areas of innovation and strategic development.

Innovative strategies to minimize immune rejection are critical for improving graft survival. This includes gene editing to generate universal donor cells and the development of novel immunosuppressive regimens that are more targeted and less systemic [22,42]. Engineering biocompatible scaffolds that precisely mimic the native corneal microenvironment can significantly enhance cell engraftment, differentiation, and organized matrix formation [50,59]. These advanced scaffolds may also serve as effective delivery vehicles for growth factors and other bioactive molecules, further promoting tissue regeneration [56,57]. Emerging characterization techniques, such as single-cell transcriptomics and proteomics, will deepen our understanding of cell behavior and differentiation pathways, thereby enhancing both safety and efficacy [15]. Gene therapy represents a promising approach for correcting underlying genetic causes of corneal disease [19,39]. Coupling gene therapy with cell transplantation holds the potential for targeted and durable treatment strategies.

Innovative in vitro and pharmacological applications are also emerging as key frontiers. Microfluidic organ-on-a-chip platforms and 3D human corneal epithelial tissue models now enable real-time assessment of barrier function, drug permeability, and toxicity, extending utility beyond graft transplantation [46,91]. Such platforms use stem cell-derived or primary corneal epithelial layers cultured under dynamic flow conditions to evaluate drug transport, TEER, and metabolic response, offering more predictive and ethically favorable alternatives to animal models. Specifically, a corneal epithelium-on-a-chip model with microengineered porous membranes and flow demonstrated functional barrier properties and drug absorption profiling under pulsatile flow, mimicking tear dynamics and enabling permeability analysis suitable for preclinical drug testing [92,93,94]. Similarly, a 3D human corneal tissue model cultured at the air–liquid interface achieved TEER values of ~1000 ± 146 Ω·cm^2^ (comparable to native tissue) and exhibited tight junction protein expression along with drug-metabolizing enzyme and transporter gene profiles—facilitating drug permeability and safety testing [95]. Conventional in vitro corneal tissue models—such as HCE-T cell-based 3D constructs—have also been widely validated for ocular toxicology and permeability assays [95].

The development of advanced preclinical models, including humanized animal models and sophisticated in vitro tissue constructs, will improve the predictive power of safety and efficacy assessments before human trials [73,96]. Furthermore, well-designed clinical trials with extended follow-up are essential to thoroughly evaluate the long-term outcomes and durability of regenerative therapies [34,78]. Moving forward, personalized therapeutic approaches tailored to an individual patient’s disease phenotype and genetic profile may further enhance treatment success. AI-powered imaging tools offer significant potential to assess graft quality, predict outcomes, and monitor long-term corneal health with unprecedented precision [29,37]. High-resolution imaging modalities, such as optical coherence tomography (OCT), will provide more accurate assessment of the severity of LSCD and provide more detailed visualization of regenerated tissue structure and function [71,97].

Finally, efforts to streamline cell culture methods, identify alternative cell sources, and optimize manufacturing workflows are essential for reducing treatment costs and improving accessibility. Comprehensive health economic evaluations will be necessary to assess the long-term value and sustainability of these advanced regenerative therapies [43,45,63,81].

## 10. Conclusions

Autologous stem cell-derived corneal epithelium with functional barrier properties holds immense promise for overcoming the limitations of current corneal reconstruction strategies. While substantial progress has been made, ongoing research is essential to ensure the safety, efficacy, and cost-effectiveness of this innovative approach. Stem cell-derived corneal epithelium has been successfully generated from a variety of sources, including ESCs, iPSCs, MSCs, LSCs, and ASCs [40,58,98]. Optimized differentiation protocols consistently reproduce critical functional features such as tight junction formation, selective permeability, and robust transepithelial electrical resistance (TEER). Advances in biocompatible scaffolds and cell-sheet engineering have further enabled the construction of functional corneal epithelium suitable for transplantation [59,99]. Autologous stem cell strategies offer significant advantages, including personalized grafts, reduced immunogenicity, and the potential to eliminate long-term immunosuppression. These cells also support wound healing and provide crucial trophic factors that aid regeneration [20,81].

Looking ahead, the future of stem cell-engineered corneal epithelium is highly promising. Nevertheless, challenges persist. Establishing standardized protocols, acquiring long-term safety data, ensuring immune compatibility, optimizing scaffold integration, and developing reproducible manufacturing processes are crucial. Additionally, improving surgical delivery methods, enhancing long-term graft performance, and guaranteeing scalability and affordability will be critical for widespread clinical adoption. Emerging innovations, such as gene-edited immune-evasive cell lines, biomimetic scaffolds, single-cell omics for fine-tuning differentiation, gene–cell combination therapies, AI-driven graft assessment, and rigorous clinical and economic evaluations, are collectively shaping a clear path toward clinically viable corneal regeneration. As stem cell biology, tissue engineering, and biomaterial science continue to converge, the potential for safe, effective, and accessible treatment for corneal blindness grows increasingly within reach.

## Figures and Tables

**Table 1:** Clinical overview of limbal stem cell transplantation (LSCT) techniques for LSCD.

Method	Indication	Donor Source	Tissue Requirement	Procedure Type	Success Rates	Advantages	Limitations
CLAU [3,15]	Unilateral LSCD	Autologous (healthy contralateral eye)	2–4 clock hours of limbus + conjunctiva	Single stage	Up to 83.2% anatomical success	No immunosuppression, long-term efficacy	Risk of iatrogenic LSCD in donor eye
CLET [4,9]	Unilateral LSCD	Autologous (small limbal biopsy)	1–2 mm^2^ biopsy	Two-stage, ex vivo expansion on amniotic membrane (GMP)	60–80%	Minimal donor tissue needed, quality control of cells	Requires cell culture facilities, increased cost
SLET [15,20]	Unilateral LSCD	Autologous (small limbal biopsy)	2–3 mm^2^ biopsy, minced	Single stage	75.2–83.8% at 1 year	Combines low donor morbidity with procedural simplicity	Long-term outcomes still under study
KLAL [15,21]	Bilateral LSCD	Allogeneic (cadaveric donor)	Large limbal segment	Single stage	67% at 12 months, 53% at 18 months	Access to LSCs when autologous source unavailable	Lifelong systemic immunosuppression, rejection risk
lr-CLAL [15]	Bilateral LSCD	Allogeneic (living relative)	Large limbal segment	Single stage	Limited data, similar to KLAL	Potential for better donor matching	Immunosuppression required, rejection risk
Allogeneic CLET [15,22]	Bilateral LSCD	Allogeneic (donor-derived cells)	Small limbal biopsy, expanded ex vivo	Two-stage, ex vivo expansion	61.4% anatomical, 53% functional success	Less immunogenic than full tissue grafts	Immunosuppression needed, limited long-term survival

Abbreviations: CLAU—conjunctival limbal autograft, CLET—cultivated autologous limbal epithelial transplantation, SLET—simple limbal epithelial transplantation, KLAL—keratolimbal allograft, lr-CLAL—living-related conjunctival limbal allograft.

**Table 2:** Stem cell types used in cornea epithelium derivation.

Classification	Cell Type	Cell Origin	Key Markers	Advantages and Outcomes	Limitations	Clinical Application
PSCs	ESCs (embryonic)	In vitro [57]	CK3, CK12, ABCG2, PAX6, p63, CD44, E-cadherin, CD271, CD29 [4,13,16,57,58]	Strong differentiation potential, full cornea formation potential [5]	Risk of uncontrolled proliferation, immune rejection tumorigenesis, ethical concerns [6]	
	iPSCs	In vitro [38]	CK3, CK12, ABCG2, PAX6, p63, CD271, CD29 [4]	Patient-specific, capacity for infinite expansion [8]	Immunological compatibility, tumorigenesis, differentiation variability [10]	Clinical study of iPSC derived corneal epithelium for transplant surgery [14]
MSCs	BMSCs	Rabbit, rat, nude rat [17]	CK3, CK12, CD271, CD29 [4,17,33]	Promotes healing, low inflammation [16]	Limited differentiation [16]	MSCs for treatment of corneal stem cell deficiency, subconjunctival injection of MSCs for LSCD [25]
	ADSCs	In vitro [18]	CK12, TGF-β, CD29 [4,40,59]	Easily accessible, low immunogenicity [16]	Limited differentiation, difficult to ensure purity [16]	
	AMSCs	Human [5]	CK3, CK12, CK18, CK19, β1-integrin, CD29 [4,25,60]	Anti-inflammatory, anti-microbial, enhances wound healing [4]	Limited source, susceptible to contamination [19]	
	UCSCs	Rabbit [18]	CK15, ABCG2, BMI1, α6 integrin, α9 integrin, β1 integrin, collagen IV, laminin, CD29 [4,18,33]	High differentiation potential, anti-inflammatory properties, low immunogenicity [5]	Limited source, difficult to culture	
ESCs (epithelial)	OMSCs	Rat, human [21]	p63, β1 integrin, collagen VII, laminin, E-cadherin, CD29 [4,43,56]	Strong regenerative potential, good epithelial integration [5]	Not cornea-specific [20]	
	ESCs (epidermal)	Goat [23]	CK3, CK12, PAX-6, CD271, CD29 [4,49]	Re-epithelialization capacity, enhanced transparency [5]	Not commonly used in ocular applications	
	HFSCs	Mouse [24]	CK12, CK15, α6 integrin, CD271, CD29 [4,49]	Proper differentiation, suppression of neovascularization	Difficult to isolate	
	LESCs	Mouse [4]	Direct differentiation into corneal epithelial cells	Native to cornea, good regenerative capacity [5]	Autologous transport is difficult [26]	Phase I/II clinical trial of cultivated autologous limbal epithelial cell [27] transplantation for LSCD
	CSCs	In vitro, human [27]	CK3, CK19, MUC5AC, Ki-67, p63, ABCG2, CD29 [4]	Proper differentiation, potential for epithelial repair [5]	LSCD patients do not have a healthy conjunctiva to harvest from [34]	

Abbreviations: PSCs—pluripotent stem cells, ESCs—embryonic stem cells, iPSCs—induced pluripotent stem cells, MSCs—mesenchymal stem cells, BMSCs—bone marrow stem cells, ADSCs—adipose-derived stem cells, AMSCs—amniotic membrane stem cells, UCSCs—umbilical cord stem cells, ESCs—epithelial stem cells, OMSCs—oral mucosa stem cells, ESCs—epidermal stem cells, HFSCs—hair follicle stem cells, LESCs—limbal epithelial stem cells, CSCs—conjunctiva stem cells.

**Table 3:** Biomaterials for corneal repair: synthetic biopolymers vs. natural biomaterials.

	Synthetic Biomaterials	Natural Biomaterials
Representative Materials	Polyethylene glycol (PEG), polylactic acid (PLA), polycaprolactone (PCL), hydrogels (e.g., PEG-based or PVA-based hydrogels) [11,27,61]	Amniotic membrane (AM), collagen, chitosan, gelatin [11,27,61]
Molecular Composition	Synthetic polymers with repeating chemical units and cross-linking agents to stabilize [11,61]	Derived from extracellular matrix proteins, glycosaminoglycans, and endogenous growth factors [27,60]
Biocompatibility	Moderate; requires chemical surface modifications (e.g., plasma treatment or coating with fibronectin/collagen) to improve cell adhesion [11]	Excellent biocompatibility due to natural ECM and growth factor composition, such as AM containing pigment epithelial derived-factor (PEDF) [27,33,34]
Transparency	High transparency, especially in hydrogels (e.g., PEG hydrogels) optimized for corneal repair [11,36]	High optical transparency; suitable for corneal epithelial healing [27]
Mechanical Properties	Tunable elasticity and strength via polymer chemistry [11,61]	Moderate mechanical strength [27,60]
Degradation Profile	Controlled degradation through tailoring polymer composition and cross-link density [27,60]	Biodegrades naturally; degradation rate may not always align with tissue healing rates [27]
Cell Adhesion	Requires bioactive coatings (e.g., RGD peptides, collagen, or fibronectin) to enhance cell attachment [11,27]	Naturally promotes corneal epithelial cell adhesion and proliferation [33,61]
Immune Response	Potential for immune response due to synthetic nature; requires biocompatible coatings [11]	Low immune response; AM contains IL-10 and TGF-β and other suppressive factors [33,34,35]
Anti-inflammatory Properties	Lacks inherent anti-inflammatory properties; external agents (e.g., corticosteroids or antimicrobial peptides) may be incorporated [11]	Possesses natural anti-inflammatory properties, reducing scarring and promoting healing (e.g., PEDF, IL-10) [33,34,35]
Antimicrobial Properties	Synthetic polymers lack inherent antimicrobial activity, but antimicrobial agents (e.g., silver nanoparticles or antibiotics) can be added [11]	Natural antimicrobial properties due to lysozyme and other bioactive molecules [27,61]
Customization	Highly customizable for mechanical strength, degradation rate, and transparency [11,60,61]	Limited customization; properties depend on donor tissue and processing methods [60]
Growth Factor Content	None; requires incorporation of exogenous growth factors (e.g., EGF) for enhanced healing [11]	AM contains intrinsic growth factors like PEDF and epidermal growth factor (EGF)
Scalability	Easily scalable; synthetic polymers can be produced in large quantities with reproducible properties [11,60]	Limited scalability; dependent on donors and tissue availability [62]
Cost	Cost-effective for large-scale production [60]	High cost due to processing, storage, and donor limitations [62]
Applications	Tissue scaffolds, drug delivery systems [11,27]	Ocular surface repair, epithelial defect healing [33,34]
Limitations	Lack native bioactivity; potential for cytotoxicity; requires external incorporation of bioactive molecules to mimic natural ECM [11,61]	Donor-dependent variability; limited shelf life; risk of disease transmission [33,62]

This table summarizes the key properties of commonly used synthetic and natural biomaterials in corneal tissue engineering, including their molecular composition, optical and mechanical properties, biocompatibility, degradation profiles, and immunomodulatory characteristics. The comparative analysis highlights the respective advantages and limitations of each material class in terms of clinical applicability, scalability, and therapeutic potential.

## Data Availability

No new data were created or analyzed in this study.
